# Characterisation of the semi-volatile component of Dissolved Organic Matter by Thermal Desorption – Proton Transfer Reaction – Mass Spectrometry

**DOI:** 10.1038/s41598-017-16256-x

**Published:** 2017-11-21

**Authors:** Dušan Materić, Mike Peacock, Matthew Kent, Sarah Cook, Vincent Gauci, Thomas Röckmann, Rupert Holzinger

**Affiliations:** 1 Faculty of Science, Institute for Marine and Atmospheric Research, Utrecht University, Princetonplein 5, 3584 CC Utrecht, Netherlands; 2 Faculty of STEM, School of Environment Earth and Ecosystems, The Open University, Milton Keynes, MK7 6AA UK; 3 Centre for Landscape & Climate Research. University of Leicester, Geography, Leicester, LE1 7RH UK; 4 Department of Aquatic Sciences and Assessment, Swedish University of Agricultural Sciences, 750 07 Uppsala, Sweden

## Abstract

Proton Transfer Reaction – Mass Spectrometry (PTR-MS) is a sensitive, soft ionisation method suitable for qualitative and quantitative analysis of volatile and semi-volatile organic vapours. PTR-MS is used for various environmental applications including monitoring of volatile organic compounds (VOCs) emitted from natural and anthropogenic sources, chemical composition measurements of aerosols, etc. Here we apply thermal desorption PTR-MS for the first time to characterise the chemical composition of dissolved organic matter (DOM). We developed a clean, low-pressure evaporation/sublimation system to remove water from samples and coupled it to a custom-made thermal desorption unit to introduce the samples to the PTR-MS. Using this system, we analysed waters from intact and degraded peat swamp forest of Kalimantan, Indonesian Borneo, and an oil palm plantation and natural forest in Sarawak, Malaysian Borneo. We detected more than 200 organic ions from these samples and principal component analysis allowed clear separation of the different sample origins based on the composition of organic compounds. The method is sensitive, reproducible, and provides a new and comparatively cheap tool for a rapid characterisation of water and soil DOM.

## Introduction

The fluvial export of dissolve organic matter DOM is a globally important process that represents a significant loss of terrestrial carbon. The biological and physico-chemical degradation of DOM in waters results in large emissions of carbon dioxide (CO_2_) to the atmosphere^1,2^. The magnitude of DOM losses can be influenced by anthropogenic disturbance acting on both large (i.e. continental) and small (i.e. catchment) scales. In addition to altering DOM quantity, both natural and anthropogenic processes can influence the composition and age of carbon exported as DOM^3–6^.

Due to the importance of DOM, numerous approaches have been used to study its composition. The simplest of these is UV-visible spectroscopy. Light absorbance at various wavelengths provides potentially useful compositional information, and 254 nm is perhaps the most popular measured wavelength^7^. In addition to absorbance, fluorescence measurements are frequently used to study DOM, generally in conjunction with excitation-emission matrices (EEMs) and parallel factor analysis (PARAFAC)^8^. This approach reveals the presence of different fractions which may be, for example, protein-like (tryptophan) or due to the presence soil fulvic acids. Complementing these simple optical measurements are simple chemical measurements. These can include measurements of hexose and pentose as indicators of whether DOM is predominantly derived from plants or microbes^9^, and a ratio of phenolic compounds to total DOM as a measure of aromaticity^10^.

Whilst the relatively simple methods above are widely used, there are also a range of analytical chemistry methods that provide information on DOM composition. For instance, high-performance size-exclusion chromatography with a UV detection wavelength of 254 nm can be used to determine DOM molecular weight^11^. Further basic information can be obtained using XAD fractionation which separates the hydrophobic and hydrophilic fractions of DOM^12^. Also, pyrolysis and thermochemolysis gas chromatography – mass spectrometry (GC-MS) have been used to identify low molecular weight decomposition products, which belong to various categories (e.g. polysaccharides, proteins, aminosugars, etc.). The exact types and proportions of decomposition products can be used as a fingerprint for different organic materials^13,14^. Ultimately, the development of high resolution mass spectrometry such as Orbitrap mass spectrometry, Quadrupole time-of-flight mass spectrometry (QTOF MS) and Fourier transform ion cyclotron resonance mass spectrometry (FT-ICR MS) has opened new fields of DOM characterisation^15,16^. FT-ICR MS has since been demonstrated to be a high resolution method for detecting individual compounds in carbon in natural waters, with one recent study identifying 4032 molecular formulae in 120 Swedish lakes^17^. FT-IRC MS however, has numerous disadvantages including: low instrument availability, high cost, the requirement for a large sample size, low time resolution, and sample preparation that requires pre-concentration and non-established routine data analysis. Subsequently, this method is both time consuming and expensive, and leads to compromises in the experimental design such as running just one replica per sample. Thus, the method may not be suitable for wide usage.

Proton Transfer Reaction – Time of Flight – Mass Spectrometry (PTR-ToF-MS) is a real-time technique achieving time resolution < 1 s, has a mass resolving power of several thousands, and has sub-ppb sensitivity^18–20^. The method utilises soft, chemical ionization by hydronium ions which results in low fragmentation of primary ions in gas stream compared to e.g. electrospray ionization. The technique allows manipulation of different energy conditions during ionization (E/N), it can use different ionization modes (H_3_O^+^, NO^+^ and O_2_^+^) and can be coupled with fastGC, which all increase analytical power at no or little time cost^21,22^.

PTR-ToF-MS has been used in many environmental studies that require high time and mass resolution such as: measurement of organic vapours concentrations/emissions in air, monitoring of oxidation processes of biogenic volatile organic compounds (BVOCs), measurement of chemical composition in organic aerosols etc.^19,23–26^. However, as the technique is developed for gaseous organic volatiles, analysis of water samples has been limited to the headspace analysis with or without a cold trap, considering equilibration between water and air^27,28^.

Here we present a rapid characterisation method of semi-volatile DOM based on PTR-ToF-MS and its first application to analyse water samples from tropical peatlands. We aimed to investigate the technique’s potential to discover novel biomarkers linked to changes in land management.

## Materials and Methods

Water samples were collected from different environments across a peat swamp forest oil palm plantation (SOP) and the surrounding natural forest buffer zone in Sarawak, Malaysian Borneo (SI); and intact, selectively logged forest (KI) and deforested, extensively drained land (KD) in Kalimantan, Indonesian Borneo. A complete list of all analysed samples is given in the supplementary material (Table S1). All samples were filtered through 0.45 μm and stored at 4 °C in the dark prior to analysis. This sampling and storage method has been shown to limit losses of total dissolved organic carbon (DOC) concentration and retain DOC composition, as measured using UV-visible spectroscopy, for periods of several months^29–31^. As the field location in Indonesian Borneo was remote and immediate refrigeration was not possible, samples were temporarily stored in the dark at ambient temperature in the coolest location at the field site (~20 °C) for a month.

For analysis of DOC concentration, samples were first filtered through 0.2 µm cellulose nitrate membrane filters. Samples were then analysed as non-purgeable organic carbon (NPOC) using a Shimadzu TOC Analyzer and appropriate standards. All sample concentrations fell within the range of standards used (0–100 mg/L).

**Figure 1:**
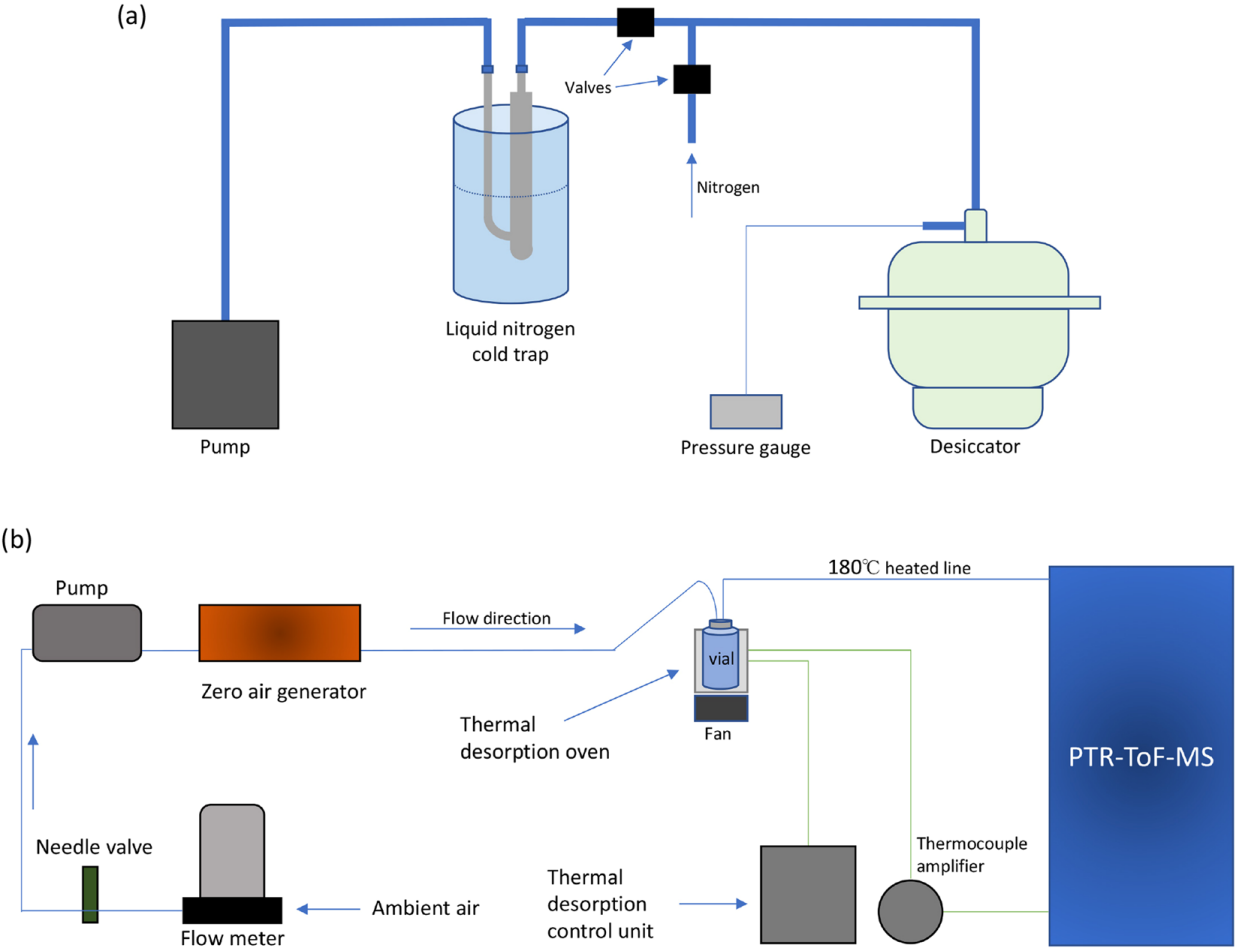
(**a**) Low pressure evaporation/sublimation system. (**b**) Thermal Desorption (TD) system coupled to PTR-ToF-MS.

A clean, low-pressure evaporation/sublimation (LPE) system was adapted in order to achieve the following goals: (1) to remove water from the samples (2) limit the loss of the organic (semi-volatile) fraction of the sample and (3) limit the sample contamination from laboratory air. We used a rotary pump to achieve suitable vacuum followed by a liquid nitrogen cold trap (to trap water from the samples as well as oil vapours from backflow of the pump) (Fig. 1a). The samples were placed in a desiccator and kept in 10 mL chromatography vials capped with Teflon caps which had two, 2 mm holes (Supplementary Fig. S1). Thus, all the internal parts of the system were glass, coated stainless steel (Restek Sulfinert) or Teflon.

The sample vials and caps were baked at 250 °C overnight. 0.5 mL of the samples (and blanks) were loaded and the vials were place in the desiccator followed by LPE, which was completed in 2 h. In order to reduce contamination between the samples and with laboratory air we re-pressurised the system by adding nitrogen slowly over 10 minutes. The vials containing the dehydrated samples were removed from the LPE system, capped with Teflon caps and analysed the next day.

The samples were loaded into a thermal desorption (TD) unit to enable transfer into the PTR-MS instrument in a clean carrier gas stream. The TD unit was designed to achieve the required temperature ramping conditions and accommodate the size of the vials (Fig. 1b). The samples were loaded in the TD oven at a temperature <35 °C, after which the following TD sequence was started: (1) 1.5 min incubation at 45 °C, (2) ramp to 220 °C at a rate of 40 °C/min, (3) 5 minutes at a constant 220 °C, and (4) cooling down to <35 °C. Additional ramping to 250 °C (after step 3) and 20 min at a constant 250 °C was done for randomly selected samples. During the TD, the samples were flushed with clean air at a flow rate of 50 mL/min as the PTR-ToF-MS was sampling.

For the measurement of organic vapours, we used a PTR-TOF 8000 (IONICON Analytik, Austria), sampling up to m/z 1130, at a time resolution of 1 spectra per second and E/N (the ratio of the electric field strength E and the gas number density N) of 129 Td.

In order to assess the contamination from different sources, we analysed system blanks (clean vials), dry blanks (clean vials exposed to LPE system together with other samples), and ultrapure water blanks (vials loaded with 0.5 mL of HPLC water, VWR chemicals), which were evaporated together with other samples in the LPE system. We measured 3 replicates of each sample to assess the reproducibility of the method.

**Figure 2:**
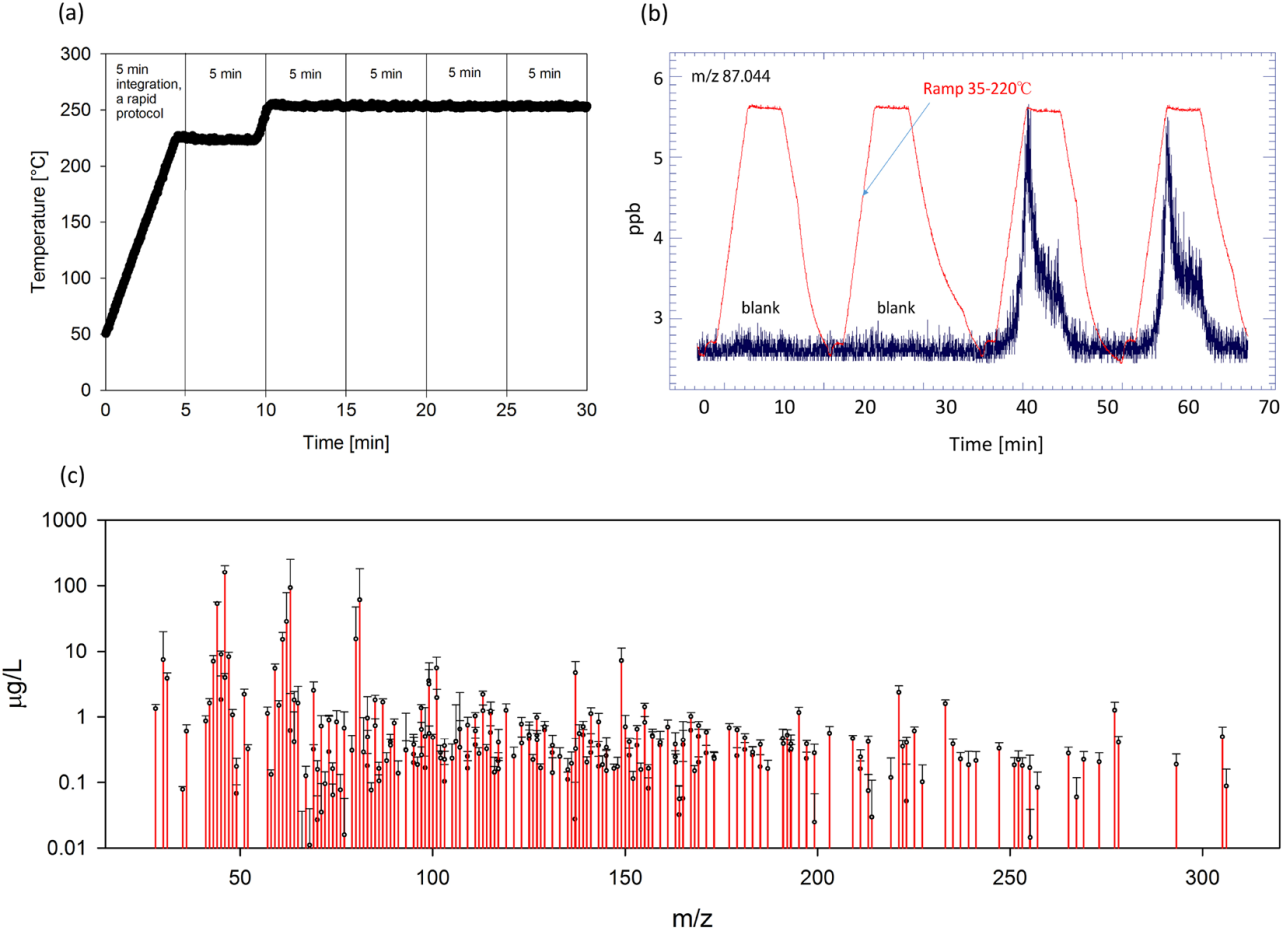
(**a**) Thermal desorption program and corresponding integration times. The rapid, 5 min heating program was used except in the long test runs. (**b**) Signal output of ion m/z 87.044 during 4 runs of thermal desorption (two blanks and two samples). (**c**) Typical mass spectrum gained after 5-minute integration (sample ID SE1W41). Note the log scale on the y-axes. Error bars present standard deviation over 7 replicates (error bars symmetrical, only top error bars plotted).

Raw PTR-ToF-MS data were analysed by PTRwid which performed peak identification and integration^32^. For each TD run, measured concentrations were integrated for 5 min starting from the point when the TD oven reached 50 °C (Fig. 2a). Figure 2b shows a typical thermogram of a TD analysis. The signal obtained from blanks measured during the same day was subtracted from each mass. The limit of detection (LoD) was established for each ion using the 3σ method, so ions signals below the LoD threshold were excluded. We compared ion signals from different groups for statistical difference by t-test and performed principal component analysis.

The chemical formula for each peak was calculated and assigned using PTRwid and cross-compared using the open source mass spectrometry tool mMass.

### Method optimization

The PTR-ToF-MS analyser is so sensitive that it detects even small impurities that originate from different sources in the system. Exposure of vials to the laboratory air was minimized because longer exposure introduced clearly measurable impurities (e.g. m/z 63 and 41). These impurities were found to be higher in dry blanks than in blanks loaded with HPLC water. This could be due to the larger glass surface area in the dry blanks compared to blanks in which the bottom of the vials were covered with water. When comparing clean vials with vials exposed to the LPE system, several impurity masses were discovered, which all contained Si atoms. We attributed this contamination to vapours originating from vacuum grease that was used to connect various parts of the system. However, these silica-based impurities are not an issue for data interpretation because they could easily be accurately identified and excluded. Nevertheless, this issue could be avoided in the future by using a vacuum seal that does not use vacuum grease.

After optimization of the LPE and TD system we performed the measurements of samples together with blanks; dry blanks and HPLC water blanks were run at the beginning and at the end of measurements, at least 2 of each per day. Samples were randomly run in 3 replicates over the course of the experiment (3 weeks).

For data analysis, we found that averaging 3 replicas for each sample, subtracting the backgrounds measured on the same day, and then averaging them assures that masses close to the detection limit are retained.

## Results and Discussion

Utilising our method, we successfully separated more than 250 organic ions ranging from 28 to 305 m/z (Fig. 2c). Ion concentrations were found to be reproducible over multiple replicates, which were run in a random order over the entire measurement. As PTR-ToF-MS is a soft ionization technique and we applied moderate temperatures during TD, we attribute most of these ions to protonated molecular ions, rather than fragments of compounds of high molecular mass. However, further experiments need to be performed to estimate potential semi-volatile ion losses due to fragmentation or LPE utilization.

The semi-volatile fraction of DOM measured with our rapid qualitative TD-PTR-ToF-MS method is 0.6% on average of the total DOM present in samples. Higher semi-volatile DOM values could be obtained with longer TD and using higher temperatures. However, high TD temperature and the increased exposure involve a risk of compound fragmentation and pyrolysis/thermolysis of high molecular masses. This would result in complex mass spectra ultimately not suitable for qualitative (compound identification) and quantitative analysis of the semi-volatile fraction of DOM, which is the main goal of this work.

**Figure 3:**
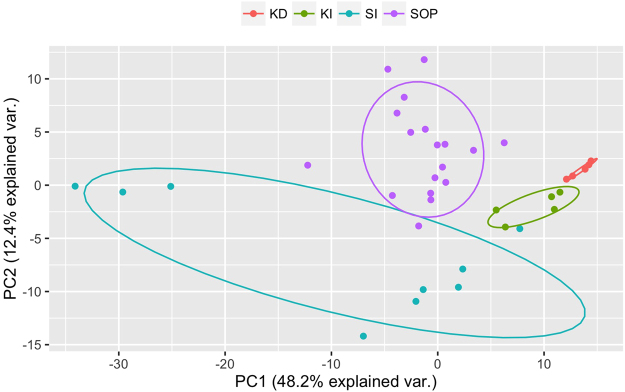
Principle component analysis of TD-PTR-ToF-MS. Samples from peat swamp forest oil palm plantation (SOP) and the surrounding natural forest buffer zones in Sarawak, Malaysian Borneo (SI); and intact, selectively logged forest (KI) and deforested, extensively drained land (KD) in Kalimantan, Indonesian Borneo.

Principal component analysis (PCA) is a useful tool for analysing and visualising complex PTR-MS data. As a first application and to demonstrate the power of this new technique, Fig. 3 shows the two first principle components resulting from a PCA of mass spectra obtained by TD-PTR-ToF-MS of water samples originating from tropical peatlands in Kalimantan and Sarawak. The data display a clear clustering related to sample origin. PC1 shows the highest values for the Kalimantan samples with slightly higher values for the degraded site. Spread around zero are samples from both Sarawak groups with the exception of three samples that have extreme negative values. These samples might have undergone slightly different chemical processes then the rest of the group, which is discussed below. PC2 shows a strong correlation with the level of the degradation/management compared to the intact state of the ecosystems, with higher values seen in the samples coming from the degraded site of Kalimantan and the palm oil plantation of Sarawak.

**Figure 4:**
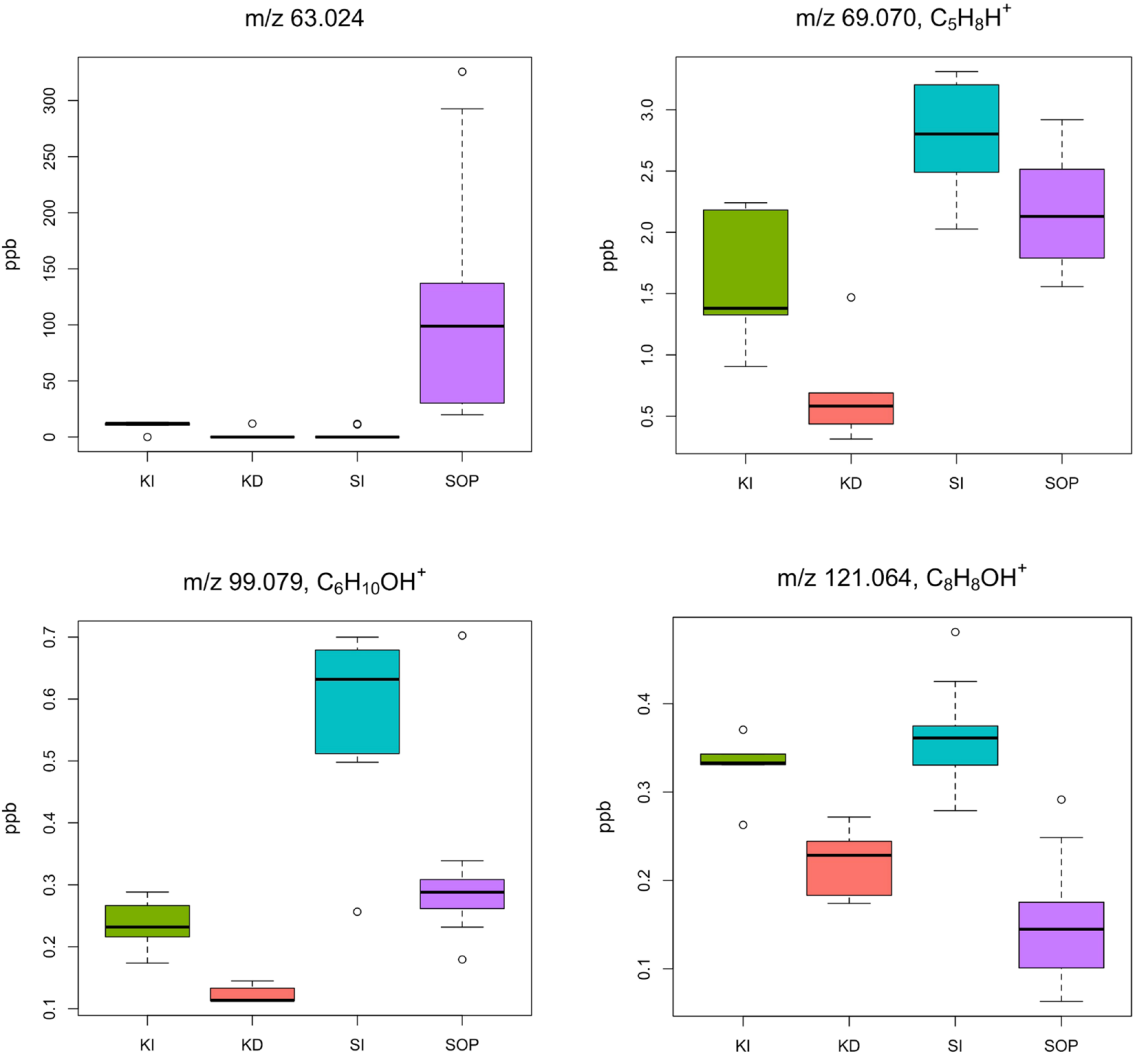
Boxplot of some significantly different organic ions (p < 0.01) with assigned chemical formula - potential biomarkers. Samples from peat swamp forest oil palm plantation (SOP) and the surrounding natural forest buffer zones in Sarawak, Malaysian Borneo (SI); and intact, selectively logged forest (KI) and deforested, extensively drained land (KD) in Kalimantan, Indonesian Borneo. The circles are presenting the most extreme data values.

**Figure 5:**
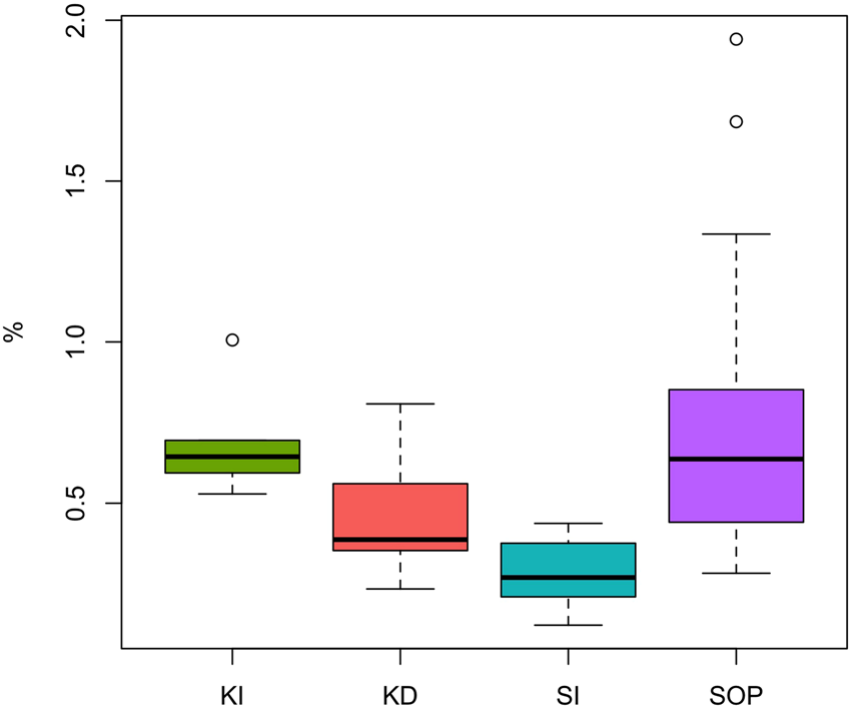
Semi-volatile fraction of DOM measured by TD-PTR-ToF-MS, normalised to the total DOM measured by a TOC Analyzer. The circles indicate the most extreme data values.

Analysis of the abundance of individual ions showed that many ions had strong significant differences (p < 0.001) between groups (Supplementary Tables S2 and S3). In most cases, the values are higher in the intact forest compared to the degraded site or oil palm ecosystems (e.g. ions m/z 69.070, 99.079, 121.064 in Fig. 4). This could be related to higher mean values of total DOC measured within intact ecosystems. However, the relative contribution of semi-volatile DOM fraction to the total DOM also showed higher values only in the intact site in Kalimantan (Fig. 5, Supplementary Table S2). This suggests that the intact ecosystem in Kalimantan possesses a higher fraction of light DOM compounds, possibly due to the rich biodiversity and complex soil biogeochemical processes^33^. On the other hand, the low fraction of semi-volatile DOM relative to the total, suggests that fluvial DOM in degraded ecosystems contains heavier, less semi-volatile compounds, which is in agreement with result shown below.

We also observed a small number of compounds which have higher values measured within the oil palm plantation (e.g. m/z 63.024, 81.036 etc.) compared to the intact site ecosystem of Sarawak (Fig. 4, Supplementary Table S3). Interestingly these compounds are highly volatile and have at least an order of magnitude higher levels at this site compared to the other locations. As their presence was only noted in oil palm plantation they might be of anthropogenic origin, possibly connected to agricultural processes or management-induced changes of the soil. However, further analysis is needed to identify these compounds and evaluate if they are biomarkers for the above mentioned conditions.

**Figure 6:**
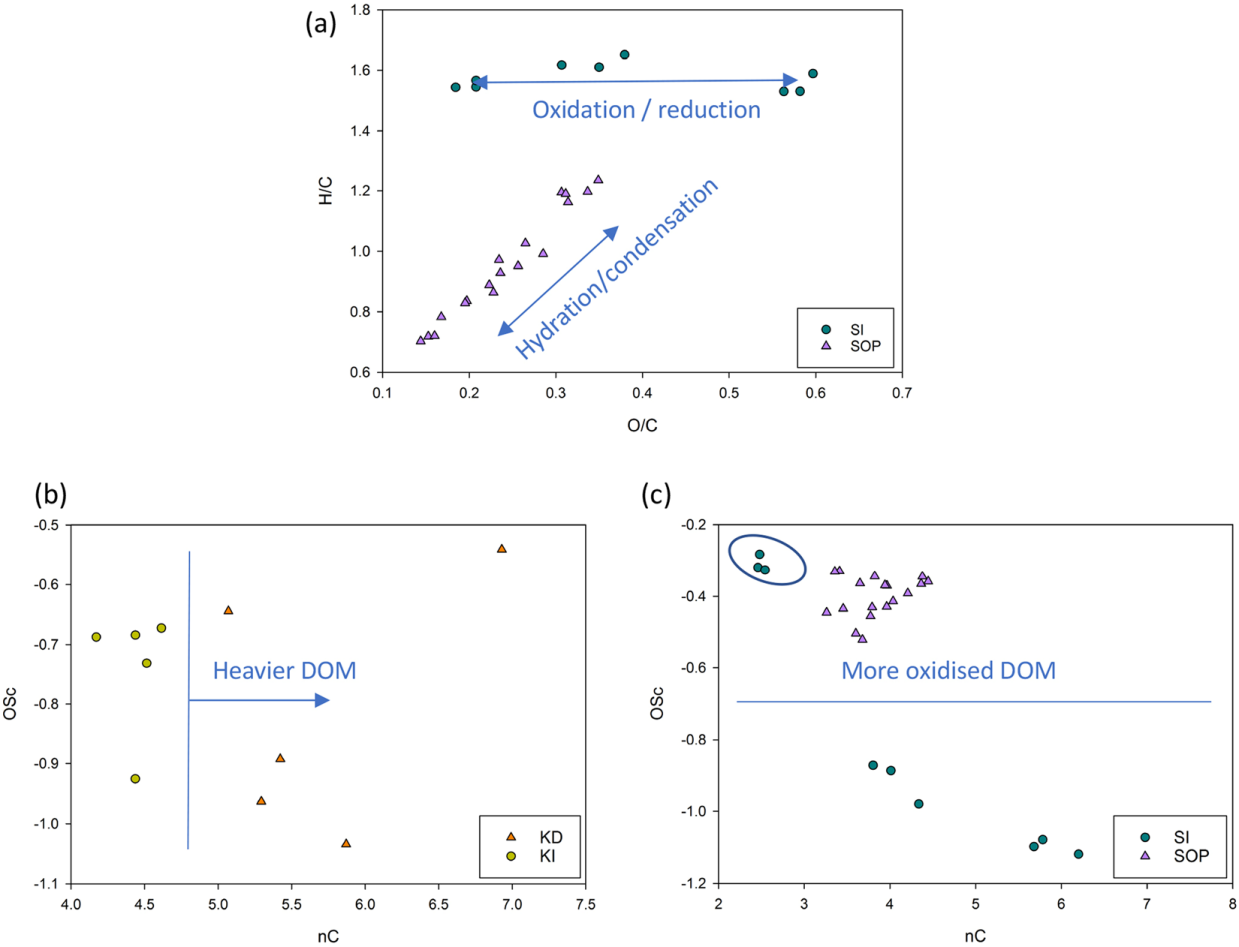
(**a**) Scatter plot of atomic ratio O/C vs H/C (Van Krevelen plot) in Sarawak samples - all ions summarised and presented as one point on the plot. (**b**) Scatter plot of chemical parameters nC and OSC for Kalimantan and (**c**) Sarawak samples.

Since the PTR-ToF-MS method is quantitative, the total number of C, H, O, N atoms can be calculated for each sample thus allowing the aromaticity index, mean oxidative state of carbon (OSc) and average number of carbon atoms per molecule in a sample (nC) to be calculated. The data can then be visualised in a matrix e.g. similar to Van Krevelen plots (Fig. 6a)^34,35^. These plots can reveal the major underlying chemical processes within the group (ecosystem). For example, Fig. 6a shows that that major processes in Sarawak’s intact forest are oxidation/reduction reactions (e.g. gaining/loosing oxygen changes O/C and not H/C), whereas in the oil palm plantation the dominant processes are hydration/condensation (e.g. gaining/losing water molecule affects both O/C and H/C). This demonstrates that different biogeochemical processes are the dominant drivers of DOM composition for these two ecosystems.

The mean oxidative state of carbon (OSc) in the Sarawak’s oil palm plantation samples is indicating overall more intense oxidation processes that might be affecting older, stored carbon (Fig. 6c). This might come as a consequence of low water table (drought stress) which is also supported by the fact that three intact forest samples had OSc values around −0.3 (Fig. 6). These samples were taken after a period of several months drought (where the water table was below –40 cm), and a period of rapid rewetting (where the water table was −13 cm). Further analysis that involved placing the drought-stressed SI samples into a separate group improved PCA clustering and revealed ions associated with the stress condition (see Supplementary Fig. S2). These ions (e.g. m/z 85.029, 97.029 and 111.045; C_4_H_4_O_2_H^+^, C_5_H_4_O_2_H^+^ and C_6_H_6_O_2_H^+^ respectively) have two oxygen atoms and can potentially be biomarkers for drought stress. The mean numbers of carbon atoms of the organic compounds analysed with PTR-ToF-MS are higher in the samples coming from Kalimantan’s degraded site compared to the intact site (Fig. 6b), which suggests presence of heavier semi-volatile DOM at this site possibly due to the mobilization of ancient carbon^36^.

However, further research needs to address the impact of the possible ion fragmentation (due to PTR or thermolysis) on O/C, H/C and OSc in order to evaluate which parameters provide a better metric to compare the results from different PTR-MS instruments and settings.

Studying DOM has become an important method of assessing the stability of carbon-rich ecosystems such as peatlands^6^. Tropical peatlands, which are found in often rapidly developing equatorial nations, are undergoing large-scale disturbances as they are converted from natural swamp forest to large-sale agricultural use^37^. For these plantations (often oil palm or paper pulp) the peatlands must be deforested and drained so that they are not waterlogged. Removing the near-constant waterlogged conditions means that the carbon-rich soils can be oxidised and leads to degradation and ecosystem instability, and studies have found that large quantities of historically stored carbon are being lost^36,38,39^. The carbon lost from the ecosystems can be rapidly converted and emitted to the atmosphere as carbon dioxide^40^. Understanding the altered carbon cycling and carbon-loss potential of degraded peatlands is therefore important to their restoration. TD-PTR-ToF-MS adds a fast and affordable technique to the biogeochemist’s analytical toolkit and allows for further interpretation the fundamental changes the ecosystems are undergoing; specific compounds (biomarkers) can be identified which could be used to elucidate process-level changes to biogeochemical interactions.

In conclusion, our rapid, high resolution TD-PTR-ToF-MS method for DOM characterisation could be potentially used both for fingerprinting approaches (untargeted) and biomarker discovery/monitoring (targeted). The advantages of our method are: (1) time resolution of a run <10 minutes, (2) small sample size <1mL, (3) it does not require sample pre-concentration, (4) it is relatively inexpensive and (5) it is both qualitative (strong analytical power) and quantitative (sub ppb levels). Further study is needed to evaluate how fragmentation during PTR and the TD affect parameters such as OSc, H/C and O/C, and to provide correction factors if needed. So far, the method is suitable for qualitative analysis of lower molecular mass groups of compounds in DOM. However, the presented method can be modified to target molecules that have higher molecular masses. Strategies such as pre-treatment of the sample, sample degradation, running in a different ionization mode and coupling to a fastGC are some of the development options that can increase sensitivity and focus the technique on specific molecular targets in a complex chemical matrix of DOM.

### Data availability

The datasets generated during and/or analysed during the current study are available from the corresponding author on reasonable request.

## Electronic supplementary material

Supplementary information

Dataset

## References

[1] Battin TJ (2009). The boundless carbon cycle. Nat. Geosci..

[2] Cole JJ (2007). Plumbing the Global Carbon Cycle: Integrating Inland Waters into the Terrestrial Carbon Budget. Ecosystems.

[3] Butman DE, Wilson HF, Barnes RT, Xenopoulos MA, Raymond PA (2015). Increased mobilization of aged carbon to rivers by human disturbance. Nat. Geosci..

[4] Evans CD (2014). Contrasting vulnerability of drained tropical and high-latitude peatlands to fluvial loss of stored carbon. Glob. Biogeochem. Cycles.

[5] Kothawala DN (2015). . The relative influence of land cover, hydrology, and in-stream processing on the composition of dissolved organic matter in boreal streams. J. Geophys. Res. Biogeosciences.

[6] Moore S (2013). Deep instability of deforested tropical peatlands revealed by fluvial organic carbon fluxes. Nature.

[7] Edzwald JK, Becker WC, Wattier KL (1985). Surrogate Parameters for Monitoring Organic Matter and THM Precursors. J. Am. Water Works Assoc..

[8] Stedmon CA, Bro R (2008). Characterizing dissolved organic matter fluorescence with parallel factor analysis: a tutorial. Limnol. Oceanogr. Methods.

[9] Strack M, Zuback Y, McCarter C, Price J (2015). Changes in dissolved organic carbon quality in soils and discharge 10 years after peatland restoration. J. Hydrol..

[10] Peacock M (2013). Quantifying dissolved organic carbon concentrations in upland catchments using phenolic proxy measurements. J. Hydrol..

[11] Zhou Q, Cabaniss SE, Maurice PA (2000). Considerations in the use of high-pressure size exclusion chromatography (HPSEC) for determining molecular weights of aquatic humic substances. Water Res..

[12] Leenheer JA (1981). Comprehensive approach to preparative isolation and fractionation of dissolved organic carbon from natural waters and wastewaters. Environ. Sci. Technol..

[13] Frazier SW (2003). Characterization of organic matter from natural waters using tetramethylammonium hydroxide thermochemolysis GC-MS. J. Anal. Appl. Pyrolysis.

[14] Leenheer JA, Croué J-P (2003). Characterizing aquatic dissolved organic matter. Environ. Sci. Technol..

[15] Hawkes JA, Dittmar T, Patriarca C, Tranvik L, Bergquist J (2016). Evaluation of the Orbitrap Mass Spectrometer for the Molecular Fingerprinting Analysis of Natural Dissolved Organic Matter. Anal. Chem..

[16] Herzsprung P (2017). Differences in DOM of rewetted and natural peatlands - Results from high-field FT-ICR-MS and bulk optical parameters. Sci. Total Environ..

[17] Kellerman AM, Dittmar T, Kothawala DN, Tranvik LJ (2014). Chemodiversity of dissolved organic matter in lakes driven by climate and hydrology. Nat. Commun..

[18] Ellis, A. M. & Mayhew, C. A. *Proton Transfer Reaction Mass Spectrometry: Principles and Applications*. (Wiley-Blackwell, 2014).

[19] Materić D (2015). Methods in Plant Foliar Volatile Organic Compounds Research. Appl. Plant Sci..

[20] Hansel A (1995). Proton transfer reaction mass spectrometry: on-line trace gas analysis at the ppb level. Int. J. Mass Spectrom. Ion Process..

[21] Jordan A (2009). An online ultra-high sensitivity Proton-transfer-reaction mass-spectrometer combined with switchable reagent ion capability (PTR + SRI − MS). Int. J. Mass Spectrom..

[22] Materić D (2015). Monoterpene separation by coupling proton transfer reaction time-of-flight mass spectrometry with fastGC. Anal. Bioanal. Chem..

[23] Holzinger R (2010). Aerosol analysis using a Thermal-Desorption Proton-Transfer-Reaction Mass Spectrometer (TD-PTR-MS): a new approach to study processing of organic aerosols. Atmos Chem Phys.

[24] Park J-H (2013). Active atmosphere-ecosystem exchange of the vast majority of detected volatile organic compounds. Science.

[25] Soukoulis C (2013). PTR-ToF-MS, A Novel, Rapid, High Sensitivity and Non-Invasive Tool to Monitor Volatile Compound Release During Fruit Post-Harvest Storage: The Case Study of Apple Ripening. Food Bioprocess Technol..

[26] Mikoviny T, Kaser L, Wisthaler A (2010). Development and characterization of a High-Temperature Proton-Transfer-Reaction Mass Spectrometer (HT-PTR-MS). Atmos Meas Tech.

[27] Kameyama S (2009). Equilibrator Inlet-Proton Transfer Reaction-Mass Spectrometry (EI-PTR-MS) for Sensitive, High-Resolution Measurement of Dimethyl Sulfide Dissolved in Seawater. Anal. Chem..

[28] Williams J (2004). Measurements of organic species in air and seawater from the tropical Atlantic. Geophys. Res. Lett..

[29] Carter HT (2012). Freshwater DOM quantity and quality from a two-component model of UV absorbance. Water Res..

[30] Cook S (2016). Cold storage as a method for the long-term preservation of tropical dissolved organic carbon (DOC). Mires Peat.

[31] Peacock M (2014). UV-visible absorbance spectroscopy as a proxy for peatland dissolved organic carbon (DOC) quantity and quality: considerations on wavelength and absorbance degradation. Environ. Sci. Process. Impacts.

[32] Holzinger R (2015). PTRwid: A new widget tool for processing PTR-TOF-MS data. Atmos Meas Tech.

[33] Ide J’ichiro (2017). Spatial variations in the molecular diversity of dissolved organic matter in water moving through a boreal forest in eastern Finland. Sci. Rep..

[34] Holzinger R, Goldstein AH, Hayes PL, Jimenez JL, Timkovsky J (2013). Chemical evolution of organic aerosol in Los Angeles during the CalNex 2010 study. Atmos Chem Phys.

[35] Kroll JH (2011). Carbon oxidation state as a metric for describing the chemistry of atmospheric organic aerosol. Nat. Chem..

[36] Miettinen J, Hooijer A, Vernimmen R, Liew SC, Page SE (2017). From carbon sink to carbon source: extensive peat oxidation in insular Southeast Asia since 1990. Environ. Res. Lett..

[37] Miettinen J (2012). Extent of industrial plantations on Southeast Asian peatlands in 2010 with analysis of historical expansion and future projections. GCB Bioenergy.

[38] Evans C (2015). Biogeochemistry: Old carbon mobilized. Nat. Geosci..

[39] Hirano T (2012). Effects of disturbances on the carbon balance of tropical peat swamp forests. Glob. Change Biol..

[40] Couwenberg J, Dommain R, Joosten H (2010). Greenhouse gas fluxes from tropical peatlands in south-east Asia. Glob. Change Biol..

