# Supplementary material for: Characterisation of the semi-volatile component of Dissolved Organic Matter by Thermal Desorption – Proton Transfer Reaction – Mass Spectrometry
Source: Sci Rep. 2017 Nov 21;7:15936. doi: 10.1038/s41598-017-16256-x (PMC5698430; doi:10.1038/s41598-017-16256-x)

Characterisation of the semi-volatile component of Dissolved Organic Matter by Thermal Desorption – Proton Transfer Reaction – Mass Spectrometry

Dušan Materić, Mike Peacock, Matthew Kent, Sarah Cook, Vincent Gauci, Thomas Röckmann and Rupert Holzinger

Table S1. List of analysed samples. Dilution 1:4 applyed for Kalimantan samples before all the experiments

RN	Samples	Type	Location	DOC (mg/L)	SVOC DOM [ug C/L]	SVOC DOM %	Group
1	F1.1 W54	Forest	Malaysia	64.94	158.83	0.24	SI
2	F1.2 W54	Forest	Malaysia	62.65	107.48	0.17	SI
3	F1.3 W54	Forest	Malaysia	66.56	80.47	0.12	SI
4	SE1 W54	Oil Palm	Malaysia	58.84	166.11	0.28	SOP
5	SE3 W54	Oil Palm	Malaysia	59.09	473.17	0.80	SOP
6	SE4 W54	Oil Palm	Malaysia	59.81	418.76	0.70	SOP
7	SA4.1 W54	Oil Palm	Malaysia	40.63	147.84	0.36	SOP
8	SA4.2 W54	Oil Palm	Malaysia	39.27	153.29	0.39	SOP
9	SA4.4 W54	Oil Palm	Malaysia	37.01	718.41	1.94	SOP
10	F1.1 W45	Forest	Malaysia	52.63	110.02	0.21	SI
11	F1.2 W45	Forest	Malaysia	52.53	173.86	0.33	SI
12	F1.3 W45	Forest	Malaysia	53.21	143.14	0.27	SI
13	SE1 W45	Oil Palm	Malaysia	45.14	174.31	0.39	SOP
14	SE3 W45	Oil Palm	Malaysia	44.27	292.07	0.66	SOP
15	SE4 W45	Oil Palm	Malaysia	47.86	806.23	1.68	SOP
16	SA4.1 W45	Oil Palm	Malaysia	38.46	198.89	0.52	SOP
17	SA4.2 W45	Oil Palm	Malaysia	38.9	181.25	0.47	SOP
18	SA4.3 W45	Oil Palm	Malaysia	40.44	178.33	0.44	SOP
19	F1.1 W41	Forest	Malaysia	92.57	347.73	0.38	SI
20	F1.2 W41	Forest	Malaysia	95.75	418.76	0.44	SI
21	F1.3 W41	Forest	Malaysia	93.26	369.17	0.40	SI
22	SE1 W41	Oil Palm	Malaysia	53.38	260.65	0.49	SOP
23	SE3 W41	Oil Palm	Malaysia	54	332.09	0.61	SOP
24	SE4 W41	Oil Palm	Malaysia	60.31	799.62	1.33	SOP
25	SA4.2 W41	Oil Palm	Malaysia	46.32	339.24	0.73	SOP
26	SA4.3 W41	Oil Palm	Malaysia	43.48	370.96	0.85	SOP
27	SA4.4 W41	Oil Palm	Malaysia	46.33	618.61	1.34	SOP
28	1107 2SEB2-D1	Intact	Borneo	13.04	90.69	0.70	KI
29	1102 2SEB2-D3	Intact	Borneo	9.96	100.19	1.01	KI
30	1059 2SEB2-DA	Intact	Borneo	10.01	64.53	0.64	KI
31	1055 2SEB2-D5	Intact	Borneo	8.32	44.00	0.53	KI
32	1104 2SEB2-D2	Intact	Borneo	10.75	63.88	0.59	KI
33	1211 2KAL1-C18	Degraded	Borneo	7.35	41.22	0.56	KD
34	1120 2KAL1-P15	Degraded	Borneo	6.38	22.51	0.35	KD
35	1239 2KAL1-P18	Degraded	Borneo	6.14	49.67	0.81	KD
36	1033 2KAL1-C15	Degraded	Borneo	5.9	13.78	0.23	KD
37	1403 2KAL1-C18E	Degraded	Borneo	6.38	24.68	0.39	KD

Characterisation of the semi-volatile component of Dissolved Organic Matter by Thermal Desorption – Proton Transfer Reaction – Mass Spectrometry

Dušan Materić, Mike Peacock, Matthew Kent, Sarah Cook, Vincent Gauci, Thomas Röckmann and Rupert Holzinger

Table S2. Ions that showed strong significant difference between intact, selectively logged forest (KI) and deforested, extensively drained land (KD) in Kalimantan, Indonesian Borneo

m/z	Chemical formula	mean KI	mean KD	p value	t value
31.017	CH2OH+	**1.68**	0.26	0.0002	-6.3
33.033	CH4OH+	**3.72**	1.12	0.0001	-7.3
42.033	C2H3NH+	**0.11**	0.00	0.0025	-6.8
45.033	C2H4OH+	**4.28**	0.00	0.0012	-8.2
47.013	CH2O2H+	**3.94**	0.00	0.0006	-9.8
51.043	?	**0.28**	0.08	0.0002	-6.3
58.03	C2H3ONH+	**0.06**	0.00	0.0048	-5.7
60.045	C2H5ONH+	**0.28**	0.03	0.0022	-4.4
65.023	CH4O3H+	**0.46**	0.00	0.0007	-9.4
67.054	C5H6H+	**0.06**	0.01	0.0020	-4.5
69.034	C4H4OH+	**0.14**	0.02	0.0014	-4.8
70.072	13CC4H8H+	**0.11**	0.02	0.0038	-4.0
85.064	C5H8OH+	**0.40**	0.18	0.0001	-7.1
89.058	C4H8O2H+	**0.27**	0.17	0.0022	-4.4
95.085	C7H10H+	**0.16**	0.07	0.0037	-4.0
97.064	C6H8OH+	**0.30**	0.15	0.0035	-4.1
97.1	C7H12H+	**0.11**	0.02	0.0014	-4.8
99.079	C6H10OH+	**0.24**	0.12	0.0007	-5.3
101.058	C5H8O2H+	**0.76**	0.45	0.0003	-6.1
109.1	C8H12H+	**0.41**	0.23	0.0036	-4.1
113.058	C6H8O2H+	**0.43**	0.22	0.0016	-4.7
117.02	C4H4O4H+	**0.10**	0.03	0.0038	-4.0
121.064	C8H8OH+	**0.33**	0.22	0.0030	-4.2
123.114	C9H14H+	**0.17**	0.10	0.0007	-5.4
127.074	C7H10O2H+	**0.18**	0.12	0.0007	-5.4
127.108	C8H14OH+	**0.20**	0.12	0.0002	-6.7
129.055	C6H8O3H+	**0.07**	0.01	0.0011	-4.9
135.045	C6H8O3H+	**0.05**	0.01	0.0034	-4.1
135.101	C6H14O3H+	**0.08**	0.00	0.0127	-4.3
141.09	C8H12O2H+	**0.31**	0.19	0.0015	-4.7
143.104	C8H12O2H+	**0.26**	0.15	0.0001	-6.9
153.089	C9H12O2H+	**0.16**	0.09	0.0035	-4.1
155.098	C9H12O2H+	**0.21**	0.13	0.0012	-4.9
171.13	C10H18O2H+	**0.11**	0.07	0.0019	-4.5
183.129	C7H18O5H+	**0.07**	0.04	0.0003	-6.2
193.136	C9H20O4H+	**0.06**	0.03	0.0023	-4.4
197.144	C12H20O2H+	**0.17**	0.14	0.0030	-4.2

Characterisation of the semi-volatile component of Dissolved Organic Matter by Thermal Desorption – Proton Transfer Reaction – Mass Spectrometry

Dušan Materić, Mike Peacock, Matthew Kent, Sarah Cook, Vincent Gauci, Thomas Röckmann and Rupert Holzinger

Table S3. Ions that showed strong significant difference between peat swamp forest oil palm plantation (SOP) and the surrounding natural forest buffer zones in Sarawak, Malaysian Borneo (SI)

**m/z**	**Chemical formula**	**mean SI**	**mean SOP**	**p value**	**t value**
41.039	C3H4H+	**2.17**	1.24	1.0E-07	7.4
45.014	?	0.00	**2.72**	1.7E-04	-4.8
51.995	?	0.01	**0.20**	3.4E-09	-8.9
62.028	?	1.64	**37.99**	3.6E-04	-4.4
63.024	DMS + ?	2.55	**117.55**	1.2E-04	-4.9
64.031	?	0.03	**0.57**	6.5E-04	-4.1
70.072	?	**0.19**	0.13	8.3E-06	5.6
71.049	C4H6OH+	**1.28**	0.53	3.8E-04	4.1
71.085	C5H10H+	**0.15**	0.06	2.0E-04	4.4
75.043	C3H6O2H+	**2.14**	0.31	1.9E-05	5.3
80.038	C4H3N2H+	0.31	**18.51**	5.6E-04	-4.2
81.035	C5H4OH+	1.27	**69.33**	3.7E-04	-4.4
85.064	C5H8OH+	**0.79**	0.41	1.2E-04	4.6
87.044	C4H6O2H+	**2.83**	0.96	2.9E-04	4.2
89.058	C4H8O2H+	**2.35**	0.38	1.2E-04	4.5
95.018	C5H2O2H+	**0.22**	0.01	1.6E-04	4.4
97.1	C7H12H+	**0.43**	0.19	4.5E-05	4.9
99.079	C6H10OH+	**0.58**	0.31	6.7E-06	5.7
103.073	C5H10O2H+	**1.12**	0.23	2.9E-04	4.2
109.065	C5H10O2H+	**0.20**	0.08	4.0E-09	8.8
111.079	C5H10O2H+	**0.31**	0.16	3.4E-05	5.0
117.089	C6H12O2H+	**0.57**	0.14	1.8E-04	4.4
121.064	C8H8OH+	**0.36**	0.15	6.4E-09	8.6
125.094	C8H12OH+	**0.43**	0.24	2.9E-05	5.1
127.074	C7H10O2H+	**0.34**	0.22	4.1E-05	5.0
127.108	C8H14OH+	**0.28**	0.18	2.2E-05	5.2
139.074	C8H10O2H+	**0.74**	0.28	4.2E-04	4.1
153.089	C9H12O2H+	**0.35**	0.23	1.9E-04	4.4
179.105	C11H14O2H+	**0.26**	0.16	1.5E-04	4.5
191.126	C9H18O4H+	**0.27**	0.10	6.8E-05	4.8
197.144	C12H20O2H+	**0.28**	0.12	1.6E-04	4.4
209.142	C9H20O5H+	**0.21**	0.12	7.0E-08	7.5
211.156	C9H22O5H+	**0.10**	0.06	4.8E-04	4.0
223.104	C16H14OH+	**0.15**	0.09	3.3E-04	4.2
235.156	C11H22O5H+	**0.13**	0.08	2.6E-05	5.1

Characterisation of the semi-volatile component of Dissolved Organic Matter by Thermal Desorption – Proton Transfer Reaction – Mass Spectrometry

Dušan Materić, Mike Peacock, Matthew Kent, Sarah Cook, Vincent Gauci, Thomas Röckmann and Rupert Holzinger

Figure S1. A picture of vials loaded with filtered samples of various color.

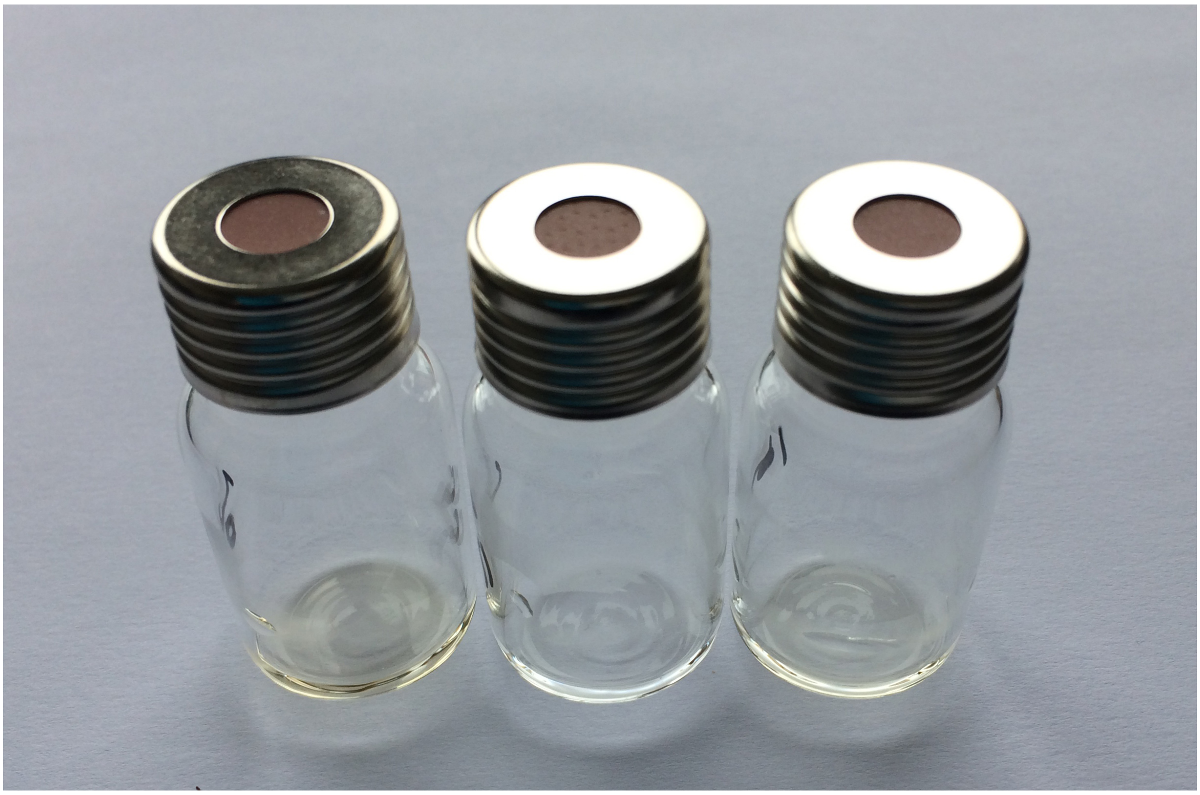

Characterisation of the semi-volatile component of Dissolved Organic Matter by Thermal Desorption – Proton Transfer Reaction – Mass Spectrometry

Dušan Materić, Mike Peacock, Matthew Kent, Sarah Cook, Vincent Gauci, Thomas Röckmann and Rupert Holzinger

Figure S2. (a, b) PCA plots of PTR-MS data, when Sarawak intact samples taken after drought-like condition were separated into a subgroup (SIs). (c, d, e) boxplot of ions associated with drought-like conditions.

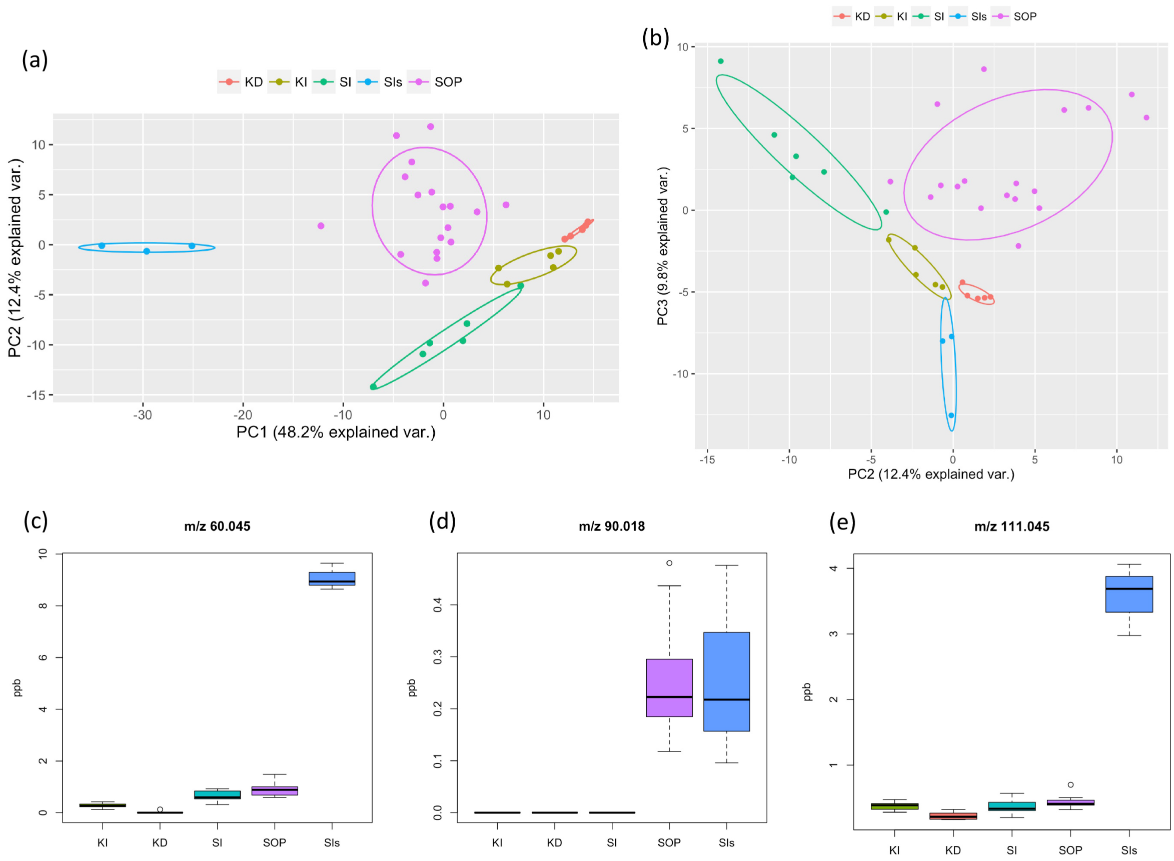

Supplement: Supplementary file 1 — Supplementary information [file 41598_2017_16256_MOESM1_ESM.pdf]
